# Acute limb ischaemia: from neglected emergency to international collaborative action

**DOI:** 10.1093/bjs/znag110

**Published:** 2026-09-10

**Authors:** Sandip Nandhra, Arun D Pherwani

**Affiliations:** Population Health Sciences Institute, Newcastle University, Newcastle-upon-Tyne, UK; Keele University School of Medicine, University Hospitals of North Midlands NHS Trust, Stoke-on-Trent, UK

Acute limb ischaemia (ALI) is one of the most devastating vascular emergencies in modern practice. Despite advances in vascular surgery, endovascular technologies, and perioperative care, patients with ALI face up to a one in five risk of major amputation and a two in five risk of death at 1 year^1^. Unlike stroke or myocardial infarction, there is little public awareness, inconsistent healthcare recognition, fragmented data capture, and substantial variation in practice.

The European multicentre PROMOTE-ALI study was an important step forwards in understanding the modern ALI population and outcomes across vascular practice. This prospective observational cohort included 705 patients from 36 vascular centres in 12 European countries, the largest prospective cohort study in ALI to date^2^. Importantly, it demonstrated that ALI is no longer simply an embolic disease of elderly patients with atrial fibrillation. Acute-on-chronic ischaemia following previous vascular intervention represented the commonest aetiology, followed by native artery thrombosis and embolic events. These findings reflect the evolving complexity of modern vascular populations, with increasing numbers of patients living longer with diffuse peripheral arterial disease, previous bypasses, stents, and hybrid reconstructions.

PROMOTE-ALI also highlighted the intricacy of decision-making in ALI. Open surgery, endovascular intervention, and hybrid procedures were all commonly used, with no significant difference in short-term amputation-free survival between strategies. This mirrors the broad therapeutic armamentarium now available to vascular specialists, but also exposes the absence of contemporary randomized evidence guiding treatment selection. Decisions are frequently influenced by the severity of ischaemia, vascular anatomy, chronicity, autogenous bypass conduit availability, patient frailty, and local expertise. The limitations of traditional classification systems such as the Rutherford grading were also apparent, particularly in predicting outcomes and guiding treatment intensity.

There is significant variation in clinical practice, usually a marker of inconsistent and low-quality clinical evidence^3^. Marked heterogeneity was observed in how clinicians approach treatment, reflecting genuine uncertainties across the specialty. Approximately half of vascular specialists favour open surgery because of concerns regarding durability, distal embolization and bleeding associated with endovascular approaches. Others use open and endovascular strategies interchangeably according to anatomy and patient factors. There is substantial variation in post-procedural antithrombotic and anticoagulation strategies, consistently highlighting the absence of clear evidence-based guidance. This represents a major unmet need in ALI care, particularly as modern interventions increasingly involve thrombectomy devices, stenting and hybrid reconstructions in patients with complex thrombotic and embolic disease.

The rapid expansion of endovascular technologies has transformed the ALI landscape. Mechanical thrombectomy devices, aspiration systems and pharmacochemical platforms are increasingly used. Recent systematic reviews demonstrate promising limb salvage and survival outcomes, with 30-day limb salvage rates exceeding 90% in contemporary series^4^. However, almost all available data remain observational, with limited direct comparison against conventional surgery. The expanding range of devices, although increasing therapeutic options, has also contributed to growing variation in practice.

The UK National Confidential Enquiry into Patient Outcome and Death (NCEPOD) report *Risking Life and Limb*^5^ exposed major system-wide failures in the delivery of ALI care. The report demonstrated substantial delays throughout the patient journey, from symptom recognition to definitive treatment. Only a minority of patients presented within the critical 6-hour window, while more than half presented over 24 h after symptom onset. Healthcare recognition was inconsistent, as was the documentation of peripheral pulses and formal Rutherford classification. Transfer delays across hub-and-spoke vascular networks were common and further contributed to treatment delays.

The report highlighted how ALI remains ‘hidden in plain sight’. Although ICD-10 provides specific codes for arterial embolism and thrombosis (such as I74.3 for the lower extremities), no single code comprehensively captures the acute limb ischaemia (ALI) syndrome across its heterogeneous aetiologies and varying pathophysiological mechanisms. This is coupled with a lack of a national registry data set capable of accurately capturing incidence, treatment patterns, or outcomes. This absence of systematic data collection is astonishing for a condition associated with such severe morbidity and mortality rates. Without robust national data infrastructure, benchmarking, quality improvement, and service planning remain limited.

These findings have catalysed a growing international movement to standardize ALI research and improve systems of care. One major challenge in ALI research has been the inconsistency of outcome reporting. Studies variably report limb salvage, death, technical success, reintervention, patency, or amputation-free survival, often at differing timepoints and with differing definitions. Patient-centred outcomes, including quality of life and functional recovery, are frequently neglected. This heterogeneity limits evidence synthesis and contributes to ongoing uncertainty regarding optimal management strategies.

The CORE-ALI initiative represents an important international response to this problem^6^. This pan-European mixed-methods study aims to develop the first Core Outcome Set (COS) for ALI using COMET and COS-STAR methodology. By incorporating perspectives from patients, clinicians, researchers, and policymakers across multiple European healthcare systems, CORE-ALI seeks to standardize outcome reporting and ensure inclusion of both clinical and patient-centred metrics. Importantly, the initiative builds directly upon lessons from PROMOTE-ALI and previous systematic reviews demonstrating substantial variability in ALI outcome reporting.

The recently funded UK NIHR ESTABLISH trial is a multicentre RCT comparing open surgical and endovascular strategies for ALI^7^. ESTABLISH is timely because much of current clinical practice is anchored on evidence generated from RCTs performed more than three decades ago, pre-dating modern surgical techniques, advanced imaging, and endovascular therapies.

Improving outcomes in ALI will require more than trials alone. The healthcare community must also address the broader systems failures highlighted within the NCEPOD report. International awareness campaigns analogous to stroke ‘FAST’ initiatives^8^ are urgently needed to improve public recognition of ALI symptoms and encourage early presentation. Greater education is required across emergency medicine, primary care, and ambulance services to reinforce ALI as a true time-critical emergency. Streamlined transfer pathways, improved imaging access, electronic record sharing, and dedicated regional ALI protocols must become standard components of vascular networks. The Vascular Society of Great Britain and Ireland has formed a working group to tackle the prescient issues with wide-ranging input from multiple healthcare specialities and patients and is on track to report before the end of 2026.

Through collaboration, standardized research frameworks, improved awareness, education, and contemporary randomized evidence, the vascular community has an opportunity to transform ALI care in the same way that coordinated systems revolutionized stroke and myocardial infarction management. Failure to do so risks continued preventable limb loss, disability, and premature death.
